# Chlorogenic Acid Ameliorates Colitis and Alters Colonic Microbiota in a Mouse Model of Dextran Sulfate Sodium-Induced Colitis

**DOI:** 10.3389/fphys.2019.00325

**Published:** 2019-03-27

**Authors:** Peng Zhang, Huanli Jiao, Chunli Wang, Yuanbang Lin, Shengyi You

**Affiliations:** Department of General Surgery, Tianjin Medical University General Hospital, Tianjin, China

**Keywords:** chlorogenic acid, colitis, MPO, TNF-α, microbiota

## Abstract

This study evaluated the mitigating effects of dietary chlorogenic acid (CGA) on colon damage and the bacterial profile in a mouse model of dextran sulfate sodium (DSS)-induced colitis. C57BL/6J mice were randomly assigned to receive one of the following treatments: (i) basal diet; (ii) basal diet with 2% CGA; (iii) basal diet with 2.5% DSS or (iv) basal diet with 2% CGA and 2.5% DSS. Following a 2-week pre-treatment period, mice in the DSS and CGA-DSS groups received 2.5% DSS in drinking water for 5 days, while the other two groups received sterile water. Compared to DSS alone, CGA was found to reduce the disease activity index, myeloperoxidase activity and tumor necrosis factor-α levels in colon tissues (*P* < 0.05). CGA also ameliorated DSS-induced inflammatory responses, reduced colon shortening and decreased the histological scores (*P* < 0.05). In an evaluation of the relative abundances of bacteria in the fecal microbiota, we found that CGA reversed the decrease in diversity caused by DSS and improved the relative abundance of organisms in the genus *Lactobacillus* (*P* < 0.05). These results indicate that CGA maintains intestinal health and reduces DSS-induced colon injury by decreasing the production of pro-inflammatory cytokines and restoring intestinal microbial diversity.

## Introduction

Inflammatory bowel disease (IBD) involves a complex and chronic inflammatory process. Although the evidence increasingly suggests that IBD results from the exposure of a genetically susceptible host to a combination of environmental factors, the exact aetiology and pathogenesis remain unclear (Tibble et al., 2000; Zhang et al., 2014). The existing evidence indicates that IBD is caused by an abnormal mucosal immune response to intestinal microorganisms and the inappropriate secretion of cytokines in the mucosa (Cario and Podolsky, 2000; Weichselbaum and Klein, 2018). Therefore, IBD treatment primarily aims to reduce recurrent inflammation and achieve a prolonged remission (Levesque et al., 2015; Sgambato et al., 2017). Traditionally, both treatment targets have been determined by clinical symptoms rather than objective evidence of inflammatory activity. However, IBD symptoms are often a direct consequence of the inflammatory process and may differ depending on the location of inflammation (Tegtmeyer et al., 2017).

Bioactive polyphenols, which have anti-oxidant and anti-inflammatory effects and can regulate cellular signaling, could potentially be used as adjuvants to the treatment of metabolic syndrome (Santino et al., 2017). Chlorogenic acid (CGA) is an important bioactive dietary phenolic substance found widely in coffee, fruits and vegetables (Naveed et al., 2018). Studies have shown that CGA has anti-oxidant, anti-bacterial and anti-inflammatory effects (Zhao et al., 2008; Kamiyama et al., 2015; Tsang et al., 2016; Zheng et al., 2016). For example, CGA inhibited the production of interleukin (IL)-8 in Caco-2 human intestinal cells in response to tumor necrosis factor (TNF)-α and H_2_O_2_, while studies in C57BL/6 mice found that CGA could alleviate dextran sulfate sodium (DSS)-induced weight loss, diarrhea, fecal blood and colon shortening. These data suggest that CGA can prevent intestinal inflammation (Shin et al., 2015). Other studies of rodent models have shown that CGA can inhibit lipopolysaccharide-induced myeloperoxidase (MPO) activity in the lung (Zatorski et al., 2015).

Colon microbes play an essential role in human health and have been associated with various diseases, including irritable bowel syndrome (Kassinen et al., 2007), autism (Li and Zhou, 2016) and obesity (Zhang et al., 2009). Most ingested CGA is not absorbed in the small intestine and reaches the colon, where it is converted into several metabolites by the local microbial community. Studies have shown that *Bifidobacterium animalis* can hydrolyse and alter the fate of CGA and thus affect the composition of the microbiota (Tomas-Barberan et al., 2014). Another study found that a 10-h course of CGA treatment increased the numbers of *Bifidobacterium* spp. and members of the *Clostridium coccoides–Eubacterium rectale* group *in vitro* and promoted the expansion of select bacteria, compared with the control group (Mills et al., 2015). Although colitis has been very well studied, evidence regarding the role of CGA in the alleviation of this disease is scarce. In this study, the effects of CGA on colonic microbial composition and pro-inflammatory factors were explored in a rodent model of DSS-induced colitis. *In vivo* experiments were also conducted to investigate whether CGA could prevent intestinal inflammation.

## Materials and Methods

### Animals and Experimental Treatments

For this study, all procedures involving animals were approved by the Animal Ethics Committee of General Hospital of Tianjin Medical University and conformed in all respects with the Guidelines for the Care and Use of Laboratory Animals of Tianjin Medical University. The female C57BL/6J mice utilized in this study were obtained from the SLAC Laboratory Animal Centre (Shanghai, China) at 6–7 weeks of age. Mice were housed in a pathogen-free colony in accordance with standard laboratory conditions, including a temperature of 22–24°C, humidity of 40–60% and 12-h daily light/dark cycle. After a 7-day adaptation period, 40 mice were randomly assigned to one of four discrete treatment groups: (1) basal diet (CON); (2) basal diet with 2% CGA (CGA); (3) basal diet with 2.5% DSS (DSS, MW 5000, KAYON Biotechnology Co., Ltd.) and (4) basal diet with 2% CGA and 2.5% DSS (CGA-DSS). CGA was dissolved in sterile drinking water for administration. All of the mice had access to food and water *ad libitum*, and the latter was changed twice weekly. During a 20-day experiment, the drinking water was supplemented or not with 2.5% DSS for the last 5 days. The body weight of each mouse was recorded at the end of the experiment.

### Sample Collection

All mice were sacrificed according to standard procedures. The colon was removed from each animal and rinsed in physiological saline to remove fecal residue. The weight and length of each organ were recorded to determine the inflammation index. Next, samples from various segments of each colon were fixed in 4% buffered formaldehyde and embedded in paraffin. Four-micrometer-thick slices of the paraffin-embedded tissues were stained with haematoxylin and eosin (H&E) in accordance with standard procedures for the histological evaluation of colonic damage. The remaining colonic tissue samples and digests were stored in frozen liquid nitrogen for further measurements of biological parameters.

### Colitis Disease Activity Index (DAI)

Tissue samples from the distal colon (5 μm) were stained with H&E and subjected to a microscopic analysis to determine the colon histological score, as proposed by Cooper et al. (1993). The following scoring system was used (maximum score = 10): 0 (rare) = severe inflammatory cell infiltration; 1 = marginally dispersed cell infiltrate; 2 = moderately increased cell infiltrate with the formation of occasional cell foci and 3 = large areas of cell infiltration causing a severe loss of tissue architecture. Additionally, the following scoring system was used to determine the extent of injury: 0 = none; 1 = mucosal; 2 = mucosal and submucosal, and 3 = transmural. Crypt damage was scored as follows: 0 = intact crypts; 1 = damage to the basal one-third; 2 = damage to the basal two-thirds damaged, 3 = only surface epithelium remains intact, and 4 = loss of entire crypt and epithelium (Backer et al., 2017).

### Assessment of Leukocyte Involvement

Myeloperoxidase activity, a marker of neutrophilic infiltration, was assessed using the method described by Grisham et al. (1990) with slight modifications. A distal colon sample from each mouse was excised, immediately rinsed with ice-cold saline, blotted dry and frozen at -70°C. Subsequently, the tissue samples were thawed, weighed and homogenized in 10 volumes of 50 mM phosphate-buffered saline (PBS; pH 7.4). The homogenates were then centrifuged at 20,000 *g* and 4°C and re-homogenized in 10 volumes of 50 mM PBS (pH 6.0) containing 0.5% hexadecyltrimethylammonium bromide (HETAB) and 10 mM ethylenediamine tetra-acetic acid (EDTA). Subsequently, the homogenates were subjected to a freeze/thaw cycle and a brief period of sonication, diluted in 50 volumes of 50 mM PBS (pH 6) and added to 50 ml of a solution containing *o*-Dianisidine dihydrochloride (0.067%), HETAB (0.5%) and hydrogen peroxide (0.003%). Complete sample reaction mixtures were placed in separate wellsand incubated in darkness for 5 min. A microplate reader (Labsystem Multiskan EX, Helsinki, Finland) was used to measure the changes in absorbance at 450 nm in accordance with the user’s manual. The results are expressed in units of U/mg protein.

### Determination of TNF-α Level

Colon samples were homogenized in ice-cold PBS as previously described and centrifuged at 3,000 *g* for 10 min. The TNF-α levels in the supernatants were determined using an enzyme-linked immunosorbent assay kit (Dou et al., 2013). Moreover, the degree of tissue inflammation was monitored using the tissue TNF-α activity level, which exhibits a linear relationship with neutrophilic infiltration in inflamed tissues. The TNF-α activity was determined in colonic samples adjacent to the installation point with a kits in accordance with the manufacturer’s instructions (CytoStore, Calgary, AB, Canada). The results from each sample are expressed in units of pg/mg of protein.

### 16S rRNA Sequencing

Thawed samples of colonic contents were mixed with PBS and homogenized using a high-speed homogenizer. The QIAamp DNA Stool Mini Kit was used to extract the total bacterial genome from each sample according to the manufacturer’s protocol (Qiagen, Hilden, Germany). A polymerase chain reaction (PCR) assay containing the Phusion^®^ High-Fidelity PCR Master Mix and GC Buffer kit (New England Biolabs, Ipswich, MA, United States) and the primers 341F (5′-CCTAYGGGRBGCASCAG-3′) and 806R (5′-GGACTACNNGGGTATCTAAT-3′) was used to amplify the 16S rRNA (V3–V4 region) genes in each sample. The following PCR conditions were applied: initial denaturation at 98°C for 30 s; 30 cycles of 98°C for 10 s, 55°C for 30 s and 72°C for 30 s and a final extension at 72°C for 5 min. The resulting PCR products were stored at 4°C until required. Later, the target fragments were detected by subjecting the PCR products to 2% agarose gel electrophoresis and were collected using the QIAquick Gel Extraction Kit (Qiagen, Germantown, MD, United States). A gene library was constructed using the Illumina TruSeq^®^ DNA PCR-Free Sample Preparation Kit (Illumina Inc., San Diego, CA, United States), quantified using Qubit and real-time PCR and sequenced on a HiSeq2500 PE250 device (Illumina Inc.).

### Statistical Analysis

SPSS, version 20 (IBM Corp., Armonk, NY, United States) was used to conduct the statistical analysis. A one-way analysis of variance with Duncan’s multiple range test was used to determine significant differences between the groups. A *P*-value of <0.05 was considered to indicate a significant difference.

## Results

### CGA Ameliorates DSS-Induced Colitis

**Figure 1:**
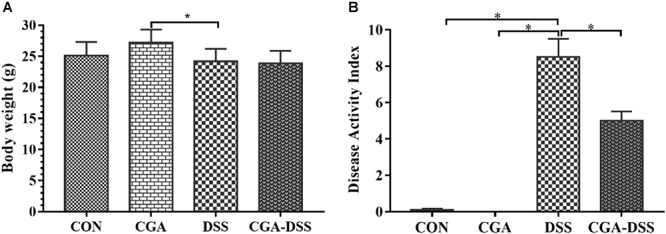
Effects of chlorogenic acid (CGA) on dextran sulfate sodium (DSS)-induced colitis. **(A)** The body weights of mice treated with CGA. **(B)** The disease activity indices (DAI) of mice after a 5-day treatment with 2.5% DSS. ^∗^indicates a *P*-value <0.05, compared with DSS.

According to the initial random group allocation, mice in the CGA and CGA-DSS groups were treated with CGA for 14 days. The other groups received the basal diet for the same length of time. Mice in the DSS and CGA-DSS groups then received 2.5% DSS for the last 5 days of the study period, after which the final body weight and DAI were recorded (Figure 1). The results demonstrate that in the CGA-DSS group dietary CGA significantly reduced the DAI, compared to the DSS group (*P* < 0.05), and alleviated DSS-induced colitis. Despite CGA failing to reverse the DSS-induced weight loss, compared with mice in the DSS group, the mice in the CGA group gained weight (*P* < 0.05).

### The Effects of CGA on MPO Activity and TNF-α Levels in Mice With DSS-Induced Colitis

**Figure 2:**
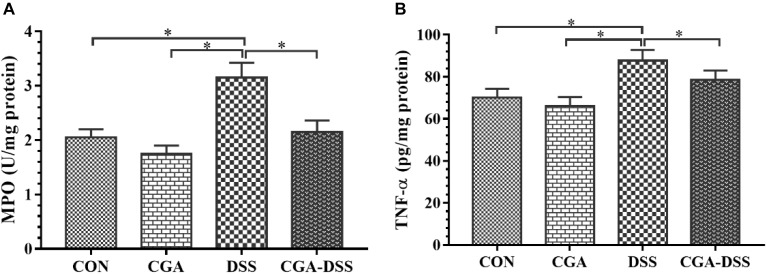
Effects of chlorogenic acid (CGA) on **(A)** myeloperoxidase (MPO) activity and **(B)** tumor necrosis factor (TNF)-α levels in a mouse model of dextran sulfate sodium (DSS)-induced colitis. ^∗^indicates a *P*-value <0.05.

Myeloperoxidase is a potential marker of tissue inflammation, tissue injury and neutrophil infiltration (Krawisz et al., 1984). The experimental results indicate increased MPO activity in the DSS group relative to the other groups (*P* < 0.05), as shown in Figure 2A. TNF-α also appears to play a significant role in the inflammatory process associated with ulcerative colitis (Moldoveanu et al., 2015). In this study, mice exposed to DSS exhibited a significant increase in TNF-α levels in the colon, whereas CGA treatment significantly mitigated this response (*P* < 0.05) as shown in Figure 2B. These data suggest that CGA exerts an anti-inflammatory effect by reducing neutrophil infiltration and pro-inflammatory cytokine production in the colon.

### The Effects of CGA on Macroscopic and Histologic Observations in DSS-Induced Colitis

**Figure 3:**
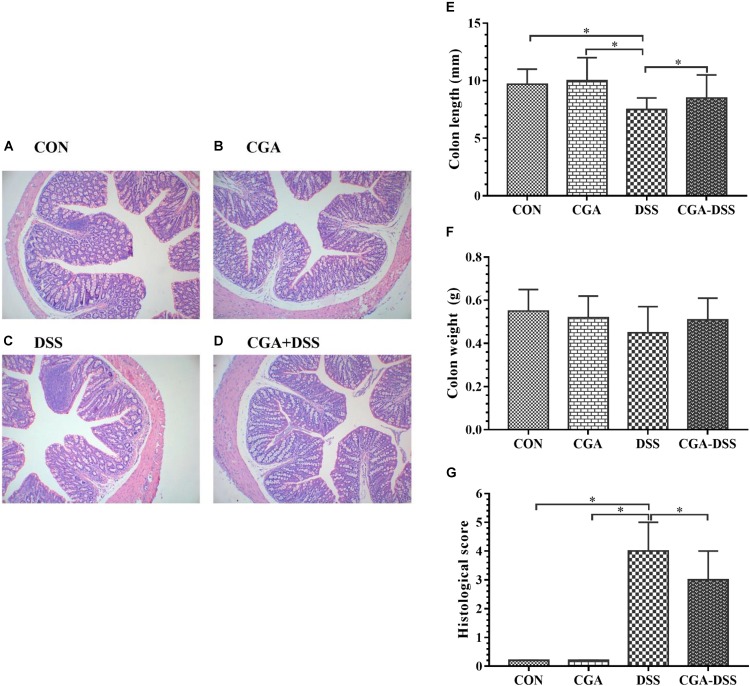
Colon tissue damage in response to dextran sulfate sodium (DSS) treatment. Effects of chlorogenic acid (CGA) on colon histology [**A**: control (CON) group, **B**: CGA group, **C**: DSS group and **D**: CGA-DSS group], colon weight **(E)**, colon length **(F)**, and histological score **(G)**. Original magnification: 100×. ^∗^indicates a *P*-value <0.05.

A histological comparison of colonic tissues from the control and DSS groups revealed multiple erosive lesions and an extensive infiltration of inflammatory cells that predominantly comprised macrophages, lymphocytes and neutrophils, along with irregular eosinophils, in the latter (Figure 3A–D). The levels of inflammatory cell infiltration and observable degree of tissue damage in the colonic tissues were assessed using the previously described histological scoring system. After a 5-day period of exposure to DSS in drinking water, mice in the DSS group exhibited an advanced inflammatory response. However, CGA had a mitigating effect on DSS-induced inflammatory markers (Figure 3G). Colon shortening is an indicator of colitis and inflammation (Siegmund, 2002). Notably, the DSS group exhibited significant colon shortening, compared to the other groups (Figure 3E, *P* < 0.05). However, no significant inter-group differences in colon weight were observed (Figure 3F).

### The Effect of CGA on Diversity in the Colonic Microbiota

**Figure 4:**
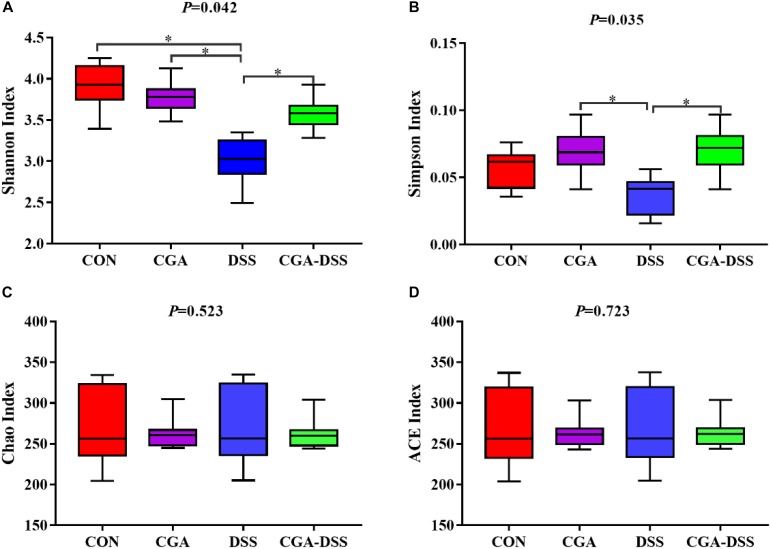
The diversity indices of bacterial communities in the mouse colon. Box plots depict differences in microbiome diversity according to the Shannon index **(A)**, Simpson index **(B)**, Chao index **(C)** and ACE index **(D)** between the control (CON), chlorogenic acid (CGA), dextran sulfate sodium (DSS) and CGA-DSS groups. ^∗^indicates a *P*-value <0.05.

Next, the V3–V4 regions of 16S rRNA isolated from the colonic digest samples were sequenced. The Shannon (Figure 4A), Simpson (Figure 4B), Chao (Figure 4C) and ACE indices (Figure 4D) were used to measure colonic microbial diversity. Treatment with DSS led to decreases in both the Shannon (*P* = 0.042) and Simpson indices (*P* = 0.035), whereas CGA treatment reversed these changes. By contrast, no significant differences were observed in the Chao and ACE indices.

### The Effect of CGA on Microbial Abundance at the Phylum Level

**Figure 5:**
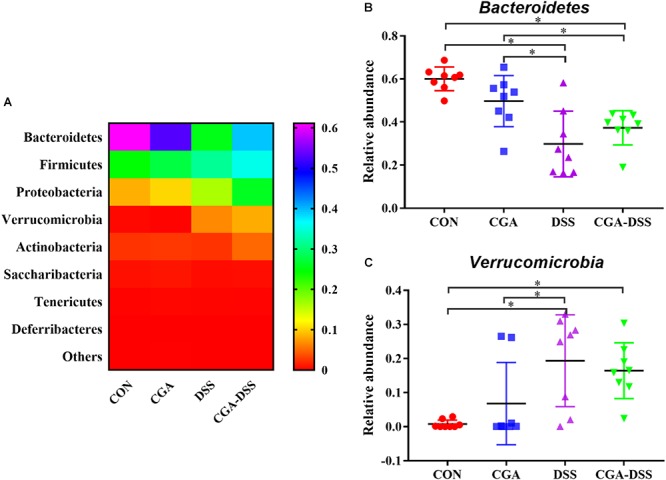
Analysis of the colon microbial composition at the phylum level. **(A)** Relative abundances of microbial phyla in the mouse colons. The relative abundances of Bacteroidetes **(B)** and Verrucomicrobia **(C)** were compared between the control (CON), chlorogenic acid (CGA), dextran sulfate sodium (DSS), and CGA-DSS groups. ^∗^indicates a *P*-value <0.05.

The major bacterial phyla in the colonic digest samples included Bacteroidetes, Firmicutes, Proteobacteria, and Verrucomicrobia, which accounted for approximately 90% of the microbiota. Bacteroidetes was most prevalent, accounting for 60.06, 49.72, 29.85, and 37.29% of the colonies in the CON, CGA, DSS, and CGA-DSS groups, respectively. The corresponding proportions of Firmicutes were 27.14, 29.45, 33.78, and 36.95%, respectively, while those of Proteobacteria were 8.62, 10.00, 8.37, and 8.20%, respectively (Figure 5A). DSS treatment decreased the relative abundance of Bacteroidetes (Figure 5B, *P* < 0.05), and this change was not reversed by CGA treatment. DSS treatment also decreased the relative abundance of Verrucomicrobia (Figure 5C).

### The Effect of CGA on Microbial Abundance at the Genus Level

**Figure 6:**
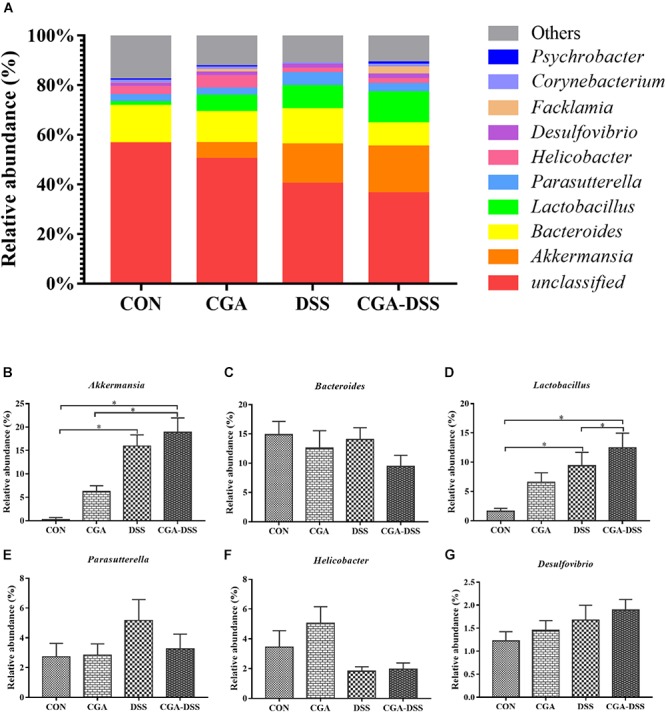
Analysis of the colon microbial composition at the genus level. **(A)** Relative abundances of microbial genera in the mouse colons. The relative abundances of *Akkermansia* **(B)**, *Bacteroides* **(C)**, *Lactobacillus* **(D)**, *Parasutterella* **(E)**, *Helicobacte*r **(F)** and *Desulfovibrio* **(G)** spp. were compared between the control (CON), chlorogenic acid (CGA), dextran sulfate sodium (DSS), and CGA-DSS groups. ^∗^indicates a *P*-value <0.05.

The 10 most abundant microbial genera are shown in Figure 6. *Bacteroides* (14.86), *Parasutterella* (2.72%), and *Helicobacter* (3.44%) were predominant in the CON group, whereas *Akkermansia*, *Bacteroides* and *Lactobacillus* were the three major strains in the CGA, DSS, and CGA-DSS groups. In the latter groups, the proportions of *Bacteroides* were 12.56, 14.05, and 9.44%, respectively, while those of *Lactobacillus* and *Akkermansia* were 6.58, 9.39, and 12.47%, respectively and 6.27, 15.94, and 18.87%, respectively. The proportion of *Akkermansia* increased in the CGA-DSS group relative to the CON and CGA groups and in the DSS group relative to the CON group. CGA also increased the relative abundance of *Lactobacillus*.

## Discussion

Continuous exposure to external stressors, such as foods, bacteria and environmental chemicals, induces a certain level of intestinal inflammation, although intestinal tissue damage and dysfunction may also play a role (Shimizu, 2017). CGA, an esterification product of caffeic acid and quinic acid, is one of the most abundant polyphenols in the human diet and has been shown to inhibit inflammatory responses in intestinal cells (Zhang et al., 2010). In this study, treatment with 2% CGA was shown to prevent weight loss and reduce the DAI. Furthermore, CGA reduced MPO activity and TNF-α levels in IBD-affected colons and ameliorated DSS-induced colon shortening and inflammatory responses. Moreover, CGA reduced the negative effects of DSS on intestinal microbial diversity and increased the relative abundance of *Lactobacillus* spp.

Intestinal dysfunction may affect the absorption of nutrients and, consequently, body weight. Furthermore, colon shortening is a sign and symptom of inflammation (Gearry et al., 2009). Studies of wild-type mice have shown that DSS-induced colitis significantly reduces body weight gain, increases epithelial permeability, rectal bleeding, colon length shortening, ulcer formation, inflammatory cell infiltration and goblet cell loss (Gadaleta et al., 2011). In a BALB/c mouse model of DSS-induced colitis, green tea polyphenols appeared to attenuate colitis by reducing the levels of TNF-α and serum amyloid A. Notably, the effects of green tea polyphenols on colitis were similar to those of sulfasalazine (Oz et al., 2013). Another study found that polyphenols from extra virgin olive oil could reduce the DAI and decrease the expression of monocyte chemoattractant protein (MCP)-1, TNF-α, cyclooxygenase (COX)-2 and inducible nitric oxide synthase (iNOS) in colon tissues when compared with DSS treatment alone. These findings suggest that dietary polyphenols may be beneficial for the treatment of ulcerative colitis (Sanchez-Fidalgo et al., 2013). Our study revealed that the addition of CGA to drinking water reduced the DAI and the severity of colon damage and shortening in response to DSS.

Cytokines are characteristic factors of IBD, wherein they act as key pathophysiological regulators of the occurrence, development and final resolution of inflammation (Strober and Fuss, 2011). In the past few decades, studies of cytokines in the context of IBD and other mucosal inflammatory conditions have been fruitful. These studies not only have provided us with important insights into the mechanisms of these diseases, but have also indicated directions for new treatment methods. Interactions between TNF family receptors and their ligands play important roles in the formation of key immune responses, including programmed cell death and lymphocyte co-stimulation (Meylan et al., 2011). CGA can inhibit the production of TNF, IL-6, INF-γ, MCP-l and macrophage inflammatory protein-l α by human peripheral blood monocytes in response to pathogenic bacteria (Krakauer, 2002). Ellagic acid was shown to reduce the expression of NF-κB, COX-2, iNOS, TNF-α, and IL-6 in 1, 2-dimethylhydrazine-induced colon cancer (Umesalma and Sudhandiran, 2010). MPO, a primarily a neutrophilic enzyme, is used as a quantitative indicator of inflammation because of the correlation between MPO activity and the histological detection of neutrophilic infiltration in the colon (Cetinkaya et al., 2006; Dost et al., 2009; Guan and Lan, 2018). In a previous rodent model study, 50 mg/kg CGA inhibited an increase in MPO activity in response to LPS and suppressed the migration of polymorphonuclear neutrophils to the lungs, as detected in bronchoalveolar lavage fluid. CGA also significantly reduced iNOS activity in lung tissues and thus inhibited the release of nitric oxide by LPS (Zhang et al., 2010). Another study found that treatment with 50 mg/kg CGA reduced the levels of MPO in gastric tissues, while a morphological analysis revealed the inhibition of neutrophilic infiltration into damaged tissues (Shimoyama et al., 2013). In this study, 2% CGA was shown to reduce MPO activity and DSS-induced damage in colon tissues.

Studies of animal models of IBD have suggested that intestinal inflammation relies heavily on a triggering event mediated by the intestinal microflora. Changes in the intestinal microflora can significantly affect both host immunity and mucosal inflammation (Hooper and Gordon, 2001; Ott et al., 2004; Huang et al., 2018; Li et al., 2018). Some changes in the microbial community are consistently observed in the context of intestinal inflammation, including reduced diversity (especially Firmicutes) and the presence of uncommon bacteria and increased concentrations of *Escherichia coli* (including pathogenic strains) (Sokol et al., 2006, 2008; Frank et al., 2007). Studies have shown that the oral administration of DSS can reduce the abundance of *Akkermansia muciniphila* and *Bacteroides acidifaciens* in feces (Kang et al., 2013; Yin et al., 2018). Recent studies of an IL10^-/-^ mouse model of IBD have identified NLRP6 as an important inhibitor of spontaneous colitis, whereas a lack of NLRP6 leads to the enrichment of *Akkermansia muciniphila* (Seregin et al., 2017; Zhang et al., 2018). Further studies of mouse models of colitis found that *Lactobacillus paracasei* could reduce intestinal inflammation and the expression of pro-inflammatory factors in the mucosa (Oliveira et al., 2011; Wu and Tian, 2017; Azad et al., 2018). Treatment with a polyphenol-rich cranberry extract was shown to significantly increase the proportion of mucus-degrading *Akkermansia* spp. in a metagenomic sample (Anhe et al., 2015). Moreover, a phenolic extract of grape pomace/grape cider (1 mg/mL) significantly increased the *Lactobacillus acidophilus* biomass in liquid medium, while exposure to the highest concentration of phenolic compounds (5000 g/disk) had no inhibitory effect on the growth of *Lactobacillus acidophilus* in an agar diffusion assay (Hervert-Hernandez et al., 2009). This study demonstrated that 2% CGA could improve microbial diversity in the colon, particularly the relative abundances of *Akkermansia* and *Lactobacillus*, and suggests that CGA could potentially restore the microecological disorder induced by DSS.

## Conclusion

In conclusion, our data provide insights into the role of CGA as a regulator of immunity and microbial diversity in the colon. Although these experiments were conducted in a mouse model of DSS-induced colitis, CGA could similarly inhibit the release of proinflammatory cytokines, such as MPO and TNF-α, in ulcerative colitis. Moreover, CGA was shown to have positive effects on the intestinal microbiota. Specifically, this polyphenol led to increases in microbial diversity and the relative abundances of *Akkermansia* and *Lactobacillus*. Our study thus demonstrates the possible use of CGA as a therapeutic adjunct to colitis treatment.

## Data Availability

All datasets generated for this study are included in the manuscript and/or the supplementary files.

## Author Contributions

PZ, HJ, CW, and YL finished all the experiments. PZ and HJ performed the statistical analysis. PZ finished the first draft of the manuscript. SY critically revised the manuscript. All the authors read and approved the manuscript.

## Conflict of Interest Statement

The authors declare that the research was conducted in the absence of any commercial or financial relationships that could be construed as a potential conflict of interest.

## References

[B1] Anhe F. F. Roy D. Pilon G. Dudonne S. Matamoros S. Varin T. V. (2015). A polyphenol-rich cranberry extract protects from diet-induced obesity, insulin resistance and intestinal inflammation in association with increased *Akkermansia* spp. population in the gut microbiota of mice. *Gut* 64 872–883. 10.1136/gutjnl-2014-307142 25080446

[B2] Azad M. A. K. Sarker M. Li T. Yin J. (2018). Probiotic species in the modulation of gut microbiota: an overview. *Biomed. Res. Int.* 2018:9478630. 10.1155/2018/9478630 29854813 PMC5964481

[B3] Backer V. Cheung F. Y. Siveke J. T. Fandrey J. Winning S. (2017). Knockdown of myeloid cell hypoxia-inducible factor-1alpha ameliorates the acute pathology in DSS-induced colitis. *PLoS One* 12:e0190074. 10.1371/journal.pone.0190074 29261815 PMC5738114

[B4] Cario E. Podolsky D. K. (2000). Differential alteration in intestinal epithelial cell expression of toll-like receptor 3 (TLR3) and TLR4 in inflammatory bowel disease. *Infect. Immun.* 68 7010–7017. 10.1128/IAI.68.12.7010-7017.2000 11083826 PMC97811

[B5] Cetinkaya A. Bulbuloglu E. Kantarceken B. Ciralik H. Kurutas E. B. Buyukbese M. A. (2006). Effects of L-carnitine on oxidant/antioxidant status in acetic acid-induced colitis. *Dig. Dis. Sci.* 51 488–494. 10.1007/s10620-006-3160-9 16614957

[B6] Cooper H. S. Murthy S. N. Shah R. S. Sedergran D. J. (1993). Clinicopathologic study of dextran sulfate sodium experimental murine colitis. *Lab. Invest.* 69 238–249. 8350599

[B7] Dost T. Ozkayran H. Gokalp F. Yenisey C. Birincioglu M. (2009). The effect of *Hypericum perforatum* (St. John’s Wort) on experimental colitis in rat. *Dig. Dis. Sci.* 54 1214–1221. 10.1007/s10620-008-0477-6 18754092

[B8] Dou W. Zhang J. Zhang E. Sun A. Ding L. Chou G. (2013). Chrysin ameliorates chemically induced colitis in the mouse through modulation of a PXR/NF-kappaB signaling pathway. *J. Pharmacol. Exp. Ther.* 345 473–482. 10.1124/jpet.112.201863 23536316 PMC3657113

[B9] Frank D. N. St Amand A. L. Feldman R. A. Boedeker E. C. Harpaz N. Pace N. R. (2007). Molecular-phylogenetic characterization of microbial community imbalances in human inflammatory bowel diseases. *Proc. Natl. Acad. Sci. U.S.A.* 104 13780–13785. 10.1073/pnas.0706625104 17699621 PMC1959459

[B10] Gadaleta R. M. van Erpecum K. J. Oldenburg B. Willemsen E. C. Renooij W. Murzilli S. (2011). Farnesoid X receptor activation inhibits inflammation and preserves the intestinal barrier in inflammatory bowel disease. *Gut* 60 463–472. 10.1136/gut.2010.212159 21242261

[B11] Gearry R. B. Irving P. M. Barrett J. S. Nathan D. M. Shepherd S. J. Gibson P. R. (2009). Reduction of dietary poorly absorbed short-chain carbohydrates (FODMAPs) improves abdominal symptoms in patients with inflammatory bowel disease-a pilot study. *J. Crohn’s Colitis* 3 8–14. 10.1016/j.crohns.2008.09.004 21172242

[B12] Grisham M. B. Benoit J. N. Granger D. N. (1990). Assessment of leukocyte involvement during ischemia and reperfusion of intestine. *Methods Enzymol.* 186 729–742. 10.1016/0076-6879(90)86172-R 2172726

[B13] Guan G. Lan S. (2018). Implications of antioxidant systems in inflammatory bowel disease. *Biomed. Res. Int.* 2018:1290179. 10.1155/2018/1290179 29854724 PMC5966678

[B14] Hervert-Hernandez D. Pintado C. Rotger R. Goni I. (2009). Stimulatory role of grape pomace polyphenols on *Lactobacillus acidophilus* growth. *Int. J. Food Microbiol.* 136 119–122. 10.1016/j.ijfoodmicro.2009.09.016 19836092

[B15] Hooper L. V. Gordon J. I. (2001). Commensal host-bacterial relationships in the gut. *Science* 292 1115–1118. 10.1126/science.1058709 11352068

[B16] Huang L. Gao R. Yu N. Zhu Y. Ding Y. Qin H. (2018). Dysbiosis of gut microbiota was closely associated with psoriasis. *Sci. China Life Sci.* 10.1007/s11427-018-9376-6 [Epub ahead of print]. 30264198

[B17] Kamiyama M. Moon J. K. Jang H. W. Shibamoto T. (2015). Role of degradation products of chlorogenic acid in the antioxidant activity of roasted coffee. *J. Agric. Food Chem.* 63 1996–2005. 10.1021/jf5060563 25658375

[B18] Kang C. S. Ban M. Choi E. J. Moon H. G. Jeon J. S. Kim D. K. (2013). Extracellular vesicles derived from gut microbiota, especially *Akkermansia muciniphila*, protect the progression of dextran sulfate sodium-induced colitis. *PLoS One* 8:e76520. 10.1371/journal.pone.0076520 24204633 PMC3811976

[B19] Kassinen A. Krogius-Kurikka L. Makivuokko H. Rinttila T. Paulin L. Corander J. (2007). The fecal microbiota of irritable bowel syndrome patients differs significantly from that of healthy subjects. *Gastroenterology* 133 24–33. 10.1053/j.gastro.2007.04.005 17631127

[B20] Krakauer T. (2002). The polyphenol chlorogenic acid inhibits staphylococcal exotoxin-induced inflammatory cytokines and chemokines. *Immunopharmacol. Immunotoxicol.* 24 113–119. 10.1081/IPH-120003407 12022439

[B21] Krawisz J. E. Sharon P. Stenson W. F. (1984). Quantitative assay for acute intestinal inflammation based on myeloperoxidase activity. assessment of inflammation in rat and hamster models. *Gastroenterology* 87 1344–1350. 6092199

[B22] Levesque B. G. Sandborn W. J. Ruel J. Feagan B. G. Sands B. E. Colombel J. F. (2015). Converging goals of treatment of inflammatory bowel disease from clinical trials and practice. *Gastroenterology* 148 37.e1–51.e1. 10.1053/j.gastro.2014.08.003 25127678

[B23] Li M. Wu Y. Hu Y. Zhao L. Zhang C. (2018). Initial gut microbiota structure affects sensitivity to DSS-induced colitis in a mouse model. *Sci. China Life Sci.* 61 762–769. 10.1007/s11427-017-9097-0 28842897

[B24] Li Q. Zhou J. M. (2016). The microbiota-gut-brain axis and its potential therapeutic role in autism spectrum disorder. *Neuroscience* 324 131–139. 10.1016/j.neuroscience.2016.03.013 26964681

[B25] Meylan F. Song Y. J. Fuss I. Villarreal S. Kahle E. Malm I. J. (2011). The TNF-family cytokine TL1A drives IL-13-dependent small intestinal inflammation. *Mucosal Immunol.* 4 172–185. 10.1038/mi.2010.67 20980995 PMC3437258

[B26] Mills C. E. Tzounis X. Oruna-Concha M. J. Mottram D. S. Gibson G. R. Spencer J. P. (2015). In vitro colonic metabolism of coffee and chlorogenic acid results in selective changes in human faecal microbiota growth. *Br. J. Nutr.* 113 1220–1227. 10.1017/S0007114514003948 25809126

[B27] Moldoveanu A. C. Diculescu M. Braticevici C. F. (2015). Cytokines in inflammatory bowel disease. *Rom. J. Intern. Med.* 53 118–127. 10.1515/rjim-2015-0016 26402980

[B28] Naveed M. Hejazi V. Abbas M. Kamboh A. A. Khan G. J. Shumzaid M. (2018). Chlorogenic acid (CGA): A pharmacological review and call for further research. *Biomed. Pharmacother.* 97 67–74. 10.1016/j.biopha.2017.10.064 29080460

[B29] Oliveira M. Bosco N. Perruisseau G. Nicolas J. Segura-Roggero I. Duboux S. (2011). *Lactobacillus paracasei* reduces intestinal inflammation in adoptive transfer mouse model of experimental colitis. *Clin. Dev. Immunol.* 2011:807483. 10.1155/2011/807483 21808650 PMC3145352

[B30] Ott S. J. Musfeldt M. Wenderoth D. F. Hampe J. Brant O. Folsch U. R. (2004). Reduction in diversity of the colonic mucosa associated bacterial microflora in patients with active inflammatory bowel disease. *Gut* 53 685–693. 10.1136/gut.2003.025403 15082587 PMC1774050

[B31] Oz H. S. Chen T. de Villiers W. J. (2013). Green tea polyphenols and sulfasalazine have parallel anti-inflammatory properties in colitis models. *Front. Immunol.* 4:132. 10.3389/fimmu.2013.00132 23761791 PMC3672863

[B32] Sanchez-Fidalgo S. Cardeno A. Sanchez-Hidalgo M. Aparicio-Soto M. de la Lastra C. A. (2013). Dietary extra virgin olive oil polyphenols supplementation modulates DSS-induced chronic colitis in mice. *J. Nutr. Biochem.* 24 1401–1413. 10.1016/j.jnutbio.2012.11.008 23337347

[B33] Santino A. Scarano A. De Santis S. De Benedictis M. Giovinazzo G. Chieppa M. (2017). Gut microbiota modulation and anti-inflammatory properties of dietary polyphenols in ibd: new and consolidated perspectives. *Curr. Pharm. Des* 23 2344–2351. 10.2174/1381612823666170207145420 28176667

[B34] Seregin S. S. Golovchenko N. Schaf B. Chen J. Pudlo N. A. Mitchell J. (2017). NLRP6 protects Il10(-/-) mice from colitis by limiting colonization of *Akkermansia muciniphila*. *Cell Rep.* 19:2174. 10.1016/j.celrep.2017.05.074 28591587

[B35] Sgambato D. Miranda A. Ranaldo R. Federico A. Romano M. (2017). The role of stress in inflammatory bowel diseases. *Curr. Pharm. Des* 23 3997–4002. 10.2174/1381612823666170228123357 28245757

[B36] Shimizu M. (2017). Multifunctions of dietary polyphenols in the regulation of intestinal inflammation. *J. Food Drug Anal.* 25 93–99. 10.1016/j.jfda.2016.12.003 28911547 PMC9333418

[B37] Shimoyama A. T. Santin J. R. Machado I. D. de Oliveira e Silva A. M. de Melo I. L. Mancini-Filho J. (2013). Antiulcerogenic activity of chlorogenic acid in different models of gastric ulcer. *Naunyn-Schmiedeberg’s Arch. Pharmacol.* 386 5–14. 10.1007/s00210-012-0807-2 23128853

[B38] Shin H. S. Satsu H. Bae M. J. Zhao Z. Ogiwara H. Totsuka M. (2015). Anti-inflammatory effect of chlorogenic acid on the IL-8 production in Caco-2 cells and the dextran sulphate sodium-induced colitis symptoms in C57BL/6 mice. *Food Chem.* 168 167–175. 10.1016/j.foodchem.2014.06.100 25172696

[B39] Siegmund B. (2002). Interleukin-1beta converting enzyme (caspase-1) in intestinal inflammation. *Biochem. Pharmacol.* 64 1–8. 10.1016/S0006-2952(02)01064-X 12106600

[B40] Sokol H. Lay C. Seksik P. Tannock G. W. (2008). Analysis of bacterial bowel communities of IBD patients: what has it revealed? *Inflamm. Bowel Dis.* 14 858–867. 10.1002/ibd.20392 18275077

[B41] Sokol H. Seksik P. Rigottier-Gois L. Lay C. Lepage P. Podglajen I. (2006). Specificities of the fecal microbiota in inflammatory bowel disease. *Inflamm. Bowel Dis.* 12 106–111. 10.1097/01.MIB.0000200323.38139.c6 16432374

[B42] Strober W. Fuss I. J. (2011). Proinflammatory cytokines in the pathogenesis of inflammatory bowel diseases. *Gastroenterology* 140 1756–1767. 10.1053/j.gastro.2011.02.016 21530742 PMC3773507

[B43] Tegtmeyer D. Seidl M. Gerner P. Baumann U. Klemann C. (2017). Inflammatory bowel disease caused by primary immunodeficiencies-Clinical presentations, review of literature, and proposal of a rational diagnostic algorithm. *Pediatr. Allergy Immunol.* 28 412–429. 10.1111/pai.12734 28513998

[B44] Tibble J. A. Sigthorsson G. Bridger S. Fagerhol M. K. Bjarnason I. (2000). Surrogate markers of intestinal inflammation are predictive of relapse in patients with inflammatory bowel disease. *Gastroenterology* 119 15–22. 10.1053/gast.2000.8523 10889150

[B45] Tomas-Barberan F. Garcia-Villalba R. Quartieri A. Raimondi S. Amaretti A. Leonardi A. (2014). In vitro transformation of chlorogenic acid by human gut microbiota. *Mol. Nutr. Food Res.* 58 1122–1131. 10.1002/mnfr.201300441 24550206

[B46] Tsang M. S. Jiao D. Chan B. C. Hon K. L. Leung P. C. Lau C. B. (2016). Anti-inflammatory activities of pentaherbs formula, berberine, gallic acid and chlorogenic acid in atopic dermatitis-like skin inflammation. *Molecules* 21:519. 10.3390/molecules21040519 27104513 PMC6274171

[B47] Umesalma S. Sudhandiran G. (2010). Differential inhibitory effects of the polyphenol ellagic acid on inflammatory mediators NF-kappaB, iNOS, COX-2, TNF-alpha, and IL-6 in 1,2-dimethylhydrazine-induced rat colon carcinogenesis. *Basic Clin. Pharmacol. Toxicol.* 107 650–655. 10.1111/j.1742-7843.2010.00565.x 20406206

[B48] Weichselbaum L. Klein O. D. (2018). The intestinal epithelial response to damage. *Sci. China Life Sci.* 61 1205–1211. 10.1007/s11427-018-9331-y 30194677

[B49] Wu X. Tian Z. (2017). Gut-liver axis: gut microbiota in shaping hepatic innate immunity. *Sci. China Life Sci.* 60 1191–1196. 10.1007/s11427-017-9128-3 28840534

[B50] Yin J. Li Y. Han H. Chen S. Gao J. Liu G. (2018). Melatonin reprogramming of gut microbiota improves lipid dysmetabolism in high-fat diet-fed mice. *J. Pineal Res.* 65:e12524. 10.1111/jpi.12524 30230594

[B51] Zatorski H. Salaga M. Zielinska M. Piechota-Polanczyk A. Owczarek K. Kordek R. (2015). Experimental colitis in mice is attenuated by topical administration of chlorogenic acid. *Naunyn-Schmiedeberg’s Arch. Pharmacol.* 388 643–651. 10.1007/s00210-015-1110-9 25743575 PMC4438256

[B52] Zhang H. DiBaise J. K. Zuccolo A. Kudrna D. Braidotti M. Yu Y. (2009). Human gut microbiota in obesity and after gastric bypass. *Proc. Natl. Acad. Sci. U.S.A.* 106 2365–2370. 10.1073/pnas.0812600106 19164560 PMC2629490

[B53] Zhang J. Dou W. Zhang E. Sun A. Ding L. Wei X. (2014). Paeoniflorin abrogates DSS-induced colitis via a TLR4-dependent pathway. *Am. J. Physiol. Gastrointest. Liver Physiol.* 306 G27–G36. 10.1152/ajpgi.00465.2012 24232001 PMC3920084

[B54] Zhang X. Huang H. Yang T. Ye Y. Shan J. Yin Z. (2010). Chlorogenic acid protects mice against lipopolysaccharide-induced acute lung injury. *Injury* 41 746–752. 10.1016/j.injury.2010.02.029 20227691

[B55] Zhang Y. Dong A. Xie K. Yu Y. (2018). Dietary supplementation with high fiber alleviates oxidative stress and inflammatory responses caused by severe sepsis in mice without altering microbiome diversity. *Front. Physiol.* 9:1929. 10.3389/fphys.2018.01929 30713502 PMC6345681

[B56] Zhao Z. Shin H. S. Satsu H. Totsuka M. Shimizu M. (2008). 5-caffeoylquinic acid and caffeic acid down-regulate the oxidative stress- and TNF-alpha-induced secretion of interleukin-8 from Caco-2 cells. *J. Agric. Food Chem.* 56 3863–3868. 10.1021/jf073168d 18444659

[B57] Zheng Y. Liu J. Cao M. L. Deng J. M. Kou J. (2016). Extrication process of chlorogenic acid in Crofton weed and antibacterial mechanism of chlorogenic acid on *Escherichia coli*. *J. Environ. Biol.* 37 1049–1055. 29989735

